# Government (Teaching) and Municipal (Non-Teaching) Hospitals

**Published:** 1912-10-12

**Authors:** Henry Burdett


### THE GERMAN AND SOME FOREIGN SYSTEMS.

### GOVERNMENT (Teaching) and MUNICIPAL (Non-Teaching) HOSPITALS.

By SIR HENRY BURDETT, K.C.B.

The proceedings at the British Hospitals Association Conference in Birmingham in relation to the National Insurance Act, with other causes, which have perturbed the hospital authorities throughout the United Kingdom, have raised problems which will have to be faced and wisely determined. A paper read by Sir William Collins on "Hospitals and the State," published in The Hospital of the 28th ultimo, page 678, usefully stated the position and various aspects of this question in relation to our British system of voluntary hospitals. It concluded with an intimation of the many difficulties which beset a State system of hospitals, and an expression of opinion that legislative experiments in relation to State interference may be overdone. His plea is, and we heartily endorse it, "that good voluntary work such as that which has been successfully organised in this country in the case of our general and special hospitals, which have supplied a felt want, have won the confidence of the public, inspired disinterested professional service among the most capable of the profession, and kept the healing of the sick—a calling almost divine—from being impersonalised and stereotyped into routine and officialdom. Such work should not be lightly thrown away through a mere careless acquiescence in a communistic trend." These are weighty words of prudent counsel, and we propose to support them by endeavouring to show, by an impartial and full account of the State and Municipal hospitals in Germany, the grounds upon which statesmen who study this question of hospital care and relief in all its bearings should hold that the people of the United Kingdom would not be content to depend in the hour of sickness upon a system of hospitals entirely conducted at the expense of the State or Municipality. There is at the present time a wide gulf between the humanitarian and divine principles upon which rests the treatment of the sick in British voluntary hospitals, and those of the foreign systems of hospitals, where science supersedes every other consideration in the conduct of the hospitals. Hence the attitude unconsciously adopted towards the sick by the majority of those who are responsible in foreign hospitals for their treatment is usually mainly or wholly scientific. No doubt great changes must come in various directions in regard to the present system of administering British hospitals under the voluntary system. Nevertheless the spirit of the voluntary system and all the great advantages and merits which it possesses must be retained. For they collectively make British hospitals undoubtedly the safest and most comfortable cure houses, from the patient's point of view, the world has ever possessed. But apart from this, as a mere matter of business, the system of voluntary hospitals throughout Great Britain should be upheld for economic reasons too, as we hope to prove in the course of the reports to which these remarks constitute an introduction.

### Hospital Systems in Europe.

Looking to the Continent of Europe we find that the hospitals in France have remained practically at a standstill, so far as reforms and developments of importance are concerned, for many years past. One result of this is that the ablest students find that they must go elsewhere than to Paris if they are to obtain an up-to-date and adequate knowledge of modern and advanced methods of treatment. Vienna used formerly to be a school to which the ablest students of other countries resorted with confidence after they had completed their medical curriculum and obtained a degree. The good reputation which Vienna formerly and properly possessed is, however, gradually being lost, because the great hospital of Vienna has long ceased to be aseptic. On hygienic grounds alone the buildings of this institution should be scrapped and a modern and up-to-date hospital erected upon a new site, where some of the highest and greatest surgeons in the world would be justified in devoting their lives to the treatment of disease. Austria sadly needs an apostle of hygiene and a disciplinarian of great authority and influence to cleanse its hospitals from the careless irregularities and personal inattention to matters of cleanliness and discipline, without which it is impossible to treat wounds with the highest efficiency and the best results. The same criticisms might be justly levied against hospitals in some other European countries, so it is not surprising that the thoroughness which characterises the work of men of science in Germany, and the great strides which that country has taken in the last twenty years especially, have brought about there a new hospital system. This system, from a scientific point of view, when seen at its best, leaves little to be desired.

### The Hospitals of Germany.

We have recently spent several weeks in Germany and have devoted much time and thought to studying and inspecting the principal hospitals there, as they are in fact at the present time. We propose in a series of reports to embody the information which we have collected, and to endeavour to bring out clearly what are the special advantages offered by the best hospitals in that country, and to what extent and where they seem to us to fail to attain to the excellence of the highest ideals of what a modern hospital can and ought to be. We wish to make it perfectly clear at the outset that in our view there are few if any hospitals in Germany, apart from the teaching hospitals, which are Governmental and Imperial institutions, and the

Stadt hospitals, which have been and are being erected throughout Germany by the municipalities in the large towns. Later we hope to give some account of a new and recent form of hospital—that provided under the National Insurance Acts by the *Kassen* or approved societies for the accommodation of their members, of which there are already upwards of fifty. We do not propose to touch the hospitals which are under the control and management of religious bodies or where they are mainly dependent upon charitable aid. This type of institution is often far behind the modern standard, and in our view, so far as Germany is concerned, such institutions are relatively of small importance and may even gradually disappear altogether in the course of years. We invite correspondence, inquiry, and criticism as these articles appear, and shall do our best to place everyone in a position to understand thoroughly every detail of the German system and to make themselves familiar with the advantages and drawbacks attaching to a system of State and Municipal hospitals conducted on the German plan. We have obtained a number of illustrations because there are many aspects of the hospitals in Germany which could not be accurately appreciated without the aid of illustrations such as those which we hope to give in the course of the present series of reports.

### No Easy Task.

It is by no means an easy task, however familiar one may be with British and other hospitals, to grasp readily and clearly the actual system of medical relief provided through the hospitals of Germany. One reason is that there no one appears to have studied the hospital system as a whole, so the general principles which underlie it cannot anywhere be ascertained, except by continuous hard work and inspection in the larger centres of population throughout Germany. This necessarily entails close and continuous labour, a great deal of travelling and infinite patience. We found, speaking broadly, that the hospital authorities were quite ready to answer questions and to give information to the best of their ability, so far as their individual department or hospital was concerned. We cannot too cordially acknowledge the great assistance which we received from a number of busy workers who went out of their way to help us to obtain the information we required and to see the hospitals and enter into their atmosphere from every point of view. There appears to be a rule, written or implied, which is strictly observed, that the officer of one hospital shall not accompany a visitor over any institution except the one to which he is himself attached. This rule is enforced even in large towns where several hospitals are established and worked by the same central authority—*i.e.*, the Magistrates' Council, for example. There is no doubt much to be said for a regulation of this kind, for it must be obvious that inconvenience and even worse things might arise if the officers in one hospital had the entrée to every other municipal hospital in the same city. It was interesting to observe, too, the sense of discipline which all hospital workers exhibit, so that they refrain from expressing an opinion or entering into questions which relate to other departments and other branches of work than their own.

### Thoroughness and Concentration.

In the best German hospitals each worker rightly concentrates all his attention upon his own field of labour and confines himself to his own department of work. This concentration of individual energy and interest makes for thoroughness and efficiency. Indeed, so far as everything connected with the patients and their treatment is concerned—that is to say, everything which relates to the work of the members of the medical profession attached to hospitals in every department in the higher type of municipal or town hospital, we doubt if efficiency could be raised to a higher point than it has reached at the present time in the best hospitals in Germany. The impression we gathered almost everywhere in this type of hospital was that not only the medical and surgical directors and their assistants, but all the many younger members of the profession working in the hospitals, do their work with a continuous efficiency as admirable as it is remarkable. The system upon which the work is organised tends to promote and insure individual efficiency. The ward work, the laboratory work, the pathological work, the work in the operation theatres, in the baths, in the gymnasium, in the special departments everywhere commences at 8 o'clock a.m. every morning and is continued until completion. Thus we found that in a big hospital like the Rudolf Virchow Krankenhaus, Berlin, and elsewhere, by half-past twelve o'clock or earlier the operations were finished and the entire operation unit was in perfect order, so that the whole of this department is left after each morning's work, however heavy the work may be, ready for the work to commence again at any moment when required. This orderly thoroughness prevailed in other hospitals, and it was easy for the trained inspector in consequence to fairly appraise the individual efficiency of those responsible for each branch of the day's work throughout the whole institution. It must be understood that in a German hospital the aim is to finish the work between the hours of 8 a.m. and 1 or 8 a.m. and 2 o'clock. Sometimes the pressure is so great as to prolong it a little, but the staff stands by the work until it is finished and then goes to the refectory. The principle everywhere seems to be first the work at its very best, and to the fullest extent the requirements of scientific treatment may demand each day, and then, but not till then, cessation. After two or two and a-half hours' interval the staff proceeds to visit the wards in many hospitals and to attend to the requirements of every patient they may contain.

### Patients well Clothed, Fed, and Provided For.

As far as our inquiries went in the best of the town or municipal hospitals, the patients were extremely well found and comfortable. The food was served in the wards hot and in a form sufficiently appetising to make it attractive to sick people. The fish, for example, was sent up to the wards steaming hot and kept so until it could be served. The bedding was good—feather pillows, horse-hair bolsters and beds, spring mattresses, iron bedsteads. Everything was of excellent quality and admirably clean and in good order. The wards are heated by hot water, and, although adequate cross-ventilation through the windows can be readily secured, there is provided ample means of artificial ventilation through electric fans usually placed in the basement, where the air is admitted from open shafts and then passed through screens over hot pipes and driven into the wards for one hour at a time, with an interval of six hours. In this way in all the pavilions the atmosphere of the wards can be completely changed, with the result that we rarely found a single ward in a Stadt hospital where the air was not pleasant and good. The exceptions were in the oldest and least efficient type of hospital, which will no doubt be torn down and rebuilt in due course.

It must be clearly understood that in what we have here written we are referring to the town or municipal hospitals and to clinical or Government hospitals in Germany where the medical staff are paid. There are also hospitals where the medical staff or some of them hold honorary office and are not necessarily bound to make their hospital duties the first consideration in their day's work. Scientific efficiency is the underlying principle which dominates the administration of the wards and every department of the best German hospitals which are connected, however remotely, with the study and treatment of disease. Seeing that such efficiency cannot be maintained where the medical staff do not commence their work early in the day, devoting themselves to it and completing it, and that where there is honorary medical service a strict adherence to hours and the commencement of the work early in the day is sometimes absent (so that the operation unit, for example, may be kept idle in the morning and then occupied, it may be, irregularly but in a general way, in the afternoon and even until a late hour), it is probable that all voluntary hospital work in German hospitals may soon be done away with. There can be no question that the best system of all is to pay all the members of the medical profession who work in hospitals, and to provide that an adequate number of them shall in the hospitals give their whole time to the work. In Germany, where this system prevails, these institutions have attained the maximum efficiency. In this type of hospital senior members of the staff—that is, the director and his assistant—frequently have the right to see private patients for a limited time each day, and they have also the right to attend and charge fees to patients in the private wards. Human nature is the same everywhere, and, as we have had instances in this country, where the general and medical committees have failed in their duty by permitting operations to proceed at irregular hours, and where it has happened that patients have been prepared for operations, kept waiting for hours, and then told that the operation would be postponed until another day. So in Germany, as our observation goes to show, similar things happen where there is voluntary service, whilst no such irregularities, not to say cruelties, do occur in the well regulated and best administered hospitals of Germany, where the whole of the medical staff are paid for their services.

### Religion Before Duty to the Patients.

In justice it must be pointed out that irregularities which do not make for the efficiency of the hospital's administration, or for the comfort and well-being of the inmates, are not confined to honorary service on the part of the medical profession either here or in Germany. In the hospitals conducted by religious bodies on certain festival and saints' days, and at other special times, it is reported that the wards are left entirely without skilled attendants, during considerable periods because the sisters place indulgence in religious ceremonies and rites before their duty and responsibility as attendants upon the sick. In London to-day we have at least one otherwise excellently administered hospital where the religious sisters absent themselves from the wards for long periods to devote themselves exclusively to their religious duties. This clear neglect of duty on the part of such sisters shows a total misapprehension of what they owe to their God as well as to their patients. Either irregularities of this kind must be eliminated by the supreme heads of the nursing sisterhoods, or, so far as efficient hospitals are concerned, the services of nuns, excellent women and trained nurses though they may be, must be discontinued in every hospital throughout the world where there is a real sense of duty and responsibility on the part of those charged with the maintenance and conduct of such institutions.

These general observations based upon knowledge we think it well to make in this introduction to a series of articles dealing with the German hospitals. Our aim and wish is to do them full justice and compare them fairly and justly with the best voluntary hospitals of which every Britisher is so proud.

### Hospital Gardens and Grounds.

Another and very pleasant side to the German hospital of the best type is the special attention which is paid to the grounds and gardens, amongst which the hospital pavilions and buildings are scattered. We propose to describe fully some of these hospital gardens and to give illustrations, for in no other way can we bring home with sufficient force the value of this particular feature to which German hospital authorities rightly attach considerable importance. Situated as some of them are in the heart of great cities, the gardens are still made most attractive by the cultivation of flowers, the careful tending of trees, grass plots, walks, and by landscape gardening most tastefully and artistically carried out, where everything has for motive the desire to make the whole aspect of the hospital garden hope-inspiring, health-giving, and grateful to the eye. Imagine, if you can, the gardens of a big hospital where birds of various kinds disport themselves at their will and pleasure, marry and are given in marriage, watch and bring up their families, and are the friends and companions of all types of men and women to be found within the hospital's boundaries. These things we have seen in Germany and have rejoiced at, and we cannot for the life of us understand why in larger hospitals, under municipal control especially, similar gardens and equal health-giving and soul-inspiring adjuncts to recovery should not be found everywhere throughout these islands, for are not our islands famed as the garden of the world?

### Whole-hearted Devotion to Work.

Another important note which won our admiration was the thoroughness with which everything is undertaken and done. It is a pleasure to realise the whole-hearted devotion, the keenness of interest, the continuous and unbroken attention which each member of the resident staff gives to his work, whether it be in the operation unit or in connection with some branch of scientific treatment, or in the pathological department, or wherever it may be. There can be no question that the residents in a German hospital are thoroughly interested in their work and do concentrate their energies upon it to the full extent of their powers. Most of them must be theoretically equipped to an extent which cannot be surpassed by any other plan of instruction. Whether the exaltation of scientific method and inquiry tends too much to theoretical perfection at the expense of practical efficiency is a question which we leave to the consideration and judgment of the working physicians, surgeons, and teachers, both here and in Germany? The Germans are conscious that this doubt has been expressed by foreign observers, but on the whole they seem inclined to adhere to the opinion that the merits of the system pursued in German hospitals in this regard warrant its continuance and justify their opinion that on the whole it is for the best.

### Some Consequences of a Rigid Discipline.

Again, the efficiency of any system, however admirable, must depend largely upon the spirit which dominates the individual men and women who bear the chief responsibility for its conduct. In Germany there is a much less keen sense of tenderness and humanity towards the sick, and, indeed, towards each other, than we, or the majority of us, in this country habitually practice. The demands of science entail discipline on the patients and the speedy carrying out of whatever has to be done in connection with their treatment. The enforcement of discipline, which is the keynote of the whole of the system of relations between the father and his family, the chief and his subordinates, emphasises the responsibility which attaches to the individual heads of every department in a German hospital. It is remarkable to notice the extraordinary deference which is displayed to the professor by all the workers in his laboratories. All this subordination to the chief is made even more striking and complete by the adoption often of an identical attitude by the chief of the professor's own staff. In such circumstances treatment *vis-à-vis* with a patient, whether it be in the operation theatre or in any other department of the hospital, must largely depend upon the attitude of mind and the character and the principles which underlie the conduct of each chief of department, throughout a great establishment, where there are sometimes as many as two thousand patients, and the immense staff required to minister to their necessities. The apprehension of these conditions and the observation of kindly forethought by some professors made us realise the actual position of each patient. The German hospital at present contains no one, as in the voluntary hospitals at home, whose special privilege and duty it is to consider the interests of the patients all the time and to minister tenderly to their comfort. Scientific treatment is apt to control affairs completely, to the setting-back of humanity and the infinite lessening of the humanising influence which the spirit of personal service has brought about, as witness the delightful atmosphere to be found in the best administered of our voluntary hospitals throughout Great Britain. It is all very well for the compulsorily insured to refuse when in hospital to be regarded as clinical material, but in practice, where science comes first, and science dominates the whole spirit of the administration of a great medical cure-house, there we may be certain that the spirit of personal service and infinite tenderness and human sympathy for the individual patient, cannot, and does not, continuously prevail.

### Protected but Not Protectors!

It has always been our assured feeling, as the result of over forty years' experience of voluntary hospitals, conducted as they are under a system which puts the interests of the patients, and not science, before every other consideration, that those who are wise will, when dangerously ill, seek treatment in a clinical hospital. We may add, however, as the result of our experience and knowledge during all these years, that no earthly consideration would induce us willingly to occupy a bed in a foreign teaching or other hospital where science was the first consideration, and dominated every act and thought of those responsible for the treatment and handling of disease in the various wards. We are speaking from the mere man's point of view, but it must be infinitely worse for every woman patient in such a hospital. This danger is one to which every statesman and every thinking man and woman in Great Britain should become fully alive, in the interests of the sick and suffering who are mainly dependent upon the hospitals when ill or when stricken by accident or other sudden disablement. The hospitals are properly the protectors of the scientific worker in the field of medicine. It follows that where an iron discipline deprives the patients in a hospital of the safeguards of an independent and articulate public opinion, there the independence of every scientific worker must be zealously protected, or the patients may be liable to a hard fate indeed. The responsibility of silence in the person of the hospital and scientific worker is a grave matter indeed, as we have often urged. State and Municipal hospitals as at present conducted, abroad especially, too often do not sufficiently safeguard the interests or adequately recognise the natural feelings of the patients. It is the system and its freedom from independent criticism, which makes possible such abuses, without remonstrance or check.

